# Corrigendum

**DOI:** 10.1080/21505594.2018.1441680

**Published:** 2018-02-23

Goldthorpe SC, Conway MJ. New insight on dengue virus-induced thrombocytopenia. Virulence. 2017;8:1492–1493

http://dx.doi.org/10.1080/21505594.2017.1368943

The article gives reference 13 instead of 12 at three places in the fourth paragraph. The correct numbering is as follows (changes in bold):

In the current study, Lin et al. (2017) suggest that DENV-envelope protein domain III (DENV-EIII) is sufficient to suppress differentiation of megakaryocytes to thrombocytes and that replication within hematopoietic precursors is not required.^12^ The investigators hypothesized that engagement of DENV-EIII with the cell surface would be sufficient to perturb cell signaling and the differentiation of the thrombocyte progenitors.**^12^** Previous research has shown that DENV antigens are present on the surface of thrombocytes and that virions can enter into these cells through engagement with DC-SIGN.^4,9^ To test their hypothesis, the investigators confirmed that DENV-EIII could bind to megakaryocytes, and then administered DENV-EIII to a mouse model and progenitor cells from both murine bone marrow and human cord blood. Treatment with DENV-EIII reduced megakaryocyte numbers in each of these model systems.**^12^** In order to understand how DENV-EIII suppressed megakaryopoiesis, megakaryocytic differentiation was performed using human cord blood-derived CD34^+^ cells. Cells treated with DENV-EIII had altered autophagy profiles and increased markers associated with apoptosis.**^12^** Previous studies have demonstrated that altered autophagy profiles can lead to cell death of megakaryocytes.^13^ These data suggest that DENV-EIII alone can modulate autophagy and induce apoptosis in progenitor and mature megakaryocytes.

The authors apologize for any inconvenience.

